# Monitoring response to neoadjuvant therapy for breast cancer in all treatment phases using an ultrasound deep learning model

**DOI:** 10.3389/fonc.2024.1255618

**Published:** 2024-01-24

**Authors:** Jingwen Zhang, Jingwen Deng, Jin Huang, Liye Mei, Ni Liao, Feng Yao, Cheng Lei, Shengrong Sun, Yimin Zhang

**Affiliations:** ^1^ Department of Breast and Thyroid Surgery, Renmin Hospital of Wuhan University, Wuhan, China; ^2^ The Institute of Technological Sciences, Wuhan University, Wuhan, China; ^3^ School of Computer Science, Hubei University of Technology, Wuhan, China; ^4^ Suzhou Institute of Wuhan University, Suzhou, China; ^5^ Shenzhen Institute of Wuhan University, Shenzhen, China

**Keywords:** breast cancer, ultrasound image, deep learning, neoadjuvant chemotherapy, pathological complete response

## Abstract

**Purpose:**

The aim of this study was to investigate the value of a deep learning model (DLM) based on breast tumor ultrasound image segmentation in predicting pathological response to neoadjuvant chemotherapy (NAC) in breast cancer.

**Methods:**

The dataset contains a total of 1393 ultrasound images of 913 patients from Renmin Hospital of Wuhan University, of which 956 ultrasound images of 856 patients were used as the training set, and 437 ultrasound images of 57 patients underwent NAC were used as the test set. A U-Net-based end-to-end DLM was developed for automatically tumor segmentation and area calculation. The predictive abilities of the DLM, manual segmentation model (MSM), and two traditional ultrasound measurement methods (longest axis model [LAM] and dual-axis model [DAM]) for pathological complete response (pCR) were compared using changes in tumor size ratios to develop receiver operating characteristic curves.

**Results:**

The average intersection over union value of the DLM was 0.856. The early-stage ultrasound-predicted area under curve (AUC) values of pCR were not significantly different from those of the intermediate and late stages (p< 0.05). The AUCs for MSM, DLM, LAM and DAM were 0.840, 0.756, 0.778 and 0.796, respectively. There was no significant difference in AUC values of the predictive ability of the four models.

**Conclusion:**

Ultrasonography was predictive of pCR in the early stages of NAC. DLM have a similar predictive value to conventional ultrasound for pCR, with an add benefit in effectively improving workflow.

## Introduction

According to the 2023 cancer statistics from the American Cancer Society, breast cancer remains the most prevalent malignant tumor worldwide, and its incidence continues to rise (1). Thus, developing treatment and evaluation strategies remains crucial. Neoadjuvant chemotherapy (NAC) represents systemic medication administered before surgical tumor excision and is a standard treatment for locally advanced breast cancer (2). NAC can downstage tumors, rendering initially inoperable tumors eligible for surgery and enhancing the breast conservation rate (3). NAC can be used to evaluate tumor response to treatment by monitoring changes in tumor size during treatment (4).

Conventional assessment for tumor response including clinical examination, pathological examination and imaging examinations. The frequency of assessment of tumor response during NAC remains controversial. The National Comprehensive Cancer Network (NCCN) guidelines advocate for routine clinical examination to assess tumor response, with imaging evaluations only warranted if tumor progression is suspected (5). However, domestic guidelines recommend that imaging evaluations should be performed at least once every two cycles (6). Pathological examination post-NAC and surgery remains the gold standard for tumor response assessment (7). Pathological complete response (pCR) is the absence of residual invasive disease in the breast and axilla (8). Patients with pCR achieve long-term disease-free survival and improved overall survival rates (9, 10). Imaging examinations such as mammography, ultrasound, and magnetic resonance imaging (MRI) are employed to evaluate patients undergoing NAC (11, 12). Mammography can serve as an effective means for the primary tumor assessment and the detection of microcalcifications. Ultrasound provides real-time monitoring, is widely accessible and cost-effective (12, 13). Contrast-enhanced MRI is considered as the most sensitive imaging modality for assessing tumor response (14).

In addition to these conventional imaging examinations, artificial intelligence (AI) has been increasingly used to automatically improve early breast cancer detection and treatment. AI algorithms such as deep learning (DL) can efficiently and automatically analyze medical images, with outstanding capabilities in locating lesions and extracting characteristic features from medical images (15). DL has been introduced to assist clinicians in breast lesion identification and segmentation, cancer grading while allowing reproducibility and visualization (16–18). AI has been applied for ultrasound assessment of NAC treatment response in breast cancer (19). Ultrasound images exhibit speckle noise, variable tumor size and shape, and tumor-like breast tissue along with echo pattern modality imaging (20), which reduce diagnostic accuracy; therefore, developing precise tumor detection algorithms that lack noise and ambiguity represents considerable challenges.

However, certain aspects that require further exploration. First, the relationship between tumor size and NAC has long been a subject of research interest. Second, deep learning algorithms based on ultrasound images for predicting NAC response can be developed (21–23). Especially for breast ultrasound image segmentation, algorithms could be categorized into two methods. One algorithm is the CNN-based networks which utilize the fixed receptive region to extract information, such as U-net (24), FCN (25) and Mask R-CNN (26). Due to the special computer kernel, the networks pay more attention to the local features (27), which work poorly in evaluating the tumor-resemble, shadows and speckle noise. Though many researchers have creatively proposed many multi-scale and attention mechanisms, the improvement is limited. The other method utilized the transformer (28, 29), which splits the ultrasound image into tokens, which employ the sequence information to acquire the global relationship from the global dimension. Therefore, the transformer would extract more features especially avoiding the interference from tumor-resemble tissue. However, because the transformer most focuses on the global features, it detects boundaries of tumor would be sensitive. To enhance the accuracy of tumor detection, the network which balances the local and global features has challenges and has the potential to widespread in radiology (30). In comparison to the conventional manual delineation of tumor regions by sonographers, this model holds advantages in terms of segmentation speed and reduction of experiential bias. Moreover, existing studies primarily focus on early-stage treatment or predicting efficacy at individual time points (31–33). The continuous evaluation of treatment response of breast cancer during the process of NAC, rather than at isolated time points, remains to be investigated.

Herein, we prospectively collected data from patients with breast cancer who underwent NAC and designed a deep learning-based tumor detection model to analyze regions of interest (ROI) in ultrasound images. Next, we verified the ability of the model to detect breast tumors without noise interference and blurry boundaries using statistical methods to monitor ultrasound images during NAC, extract information from feature maps, and provide an intuitive and quantitative picture of tumor alterations. Furthermore, we examined the performance of proportional changes in tumor size measured using conventional models and DLM for predicting the response to chemotherapy. We employed DLM and conventional ultrasound throughout all phases of the NAC treatment to monitor its efficacy. The study aimed to assess the ability of DLM in predicting pCR in breast cancer patients undergoing NAC and its ability to evaluate the treatment response at various stages of NAC.

## Methods

### Patients

**Figure 1:**
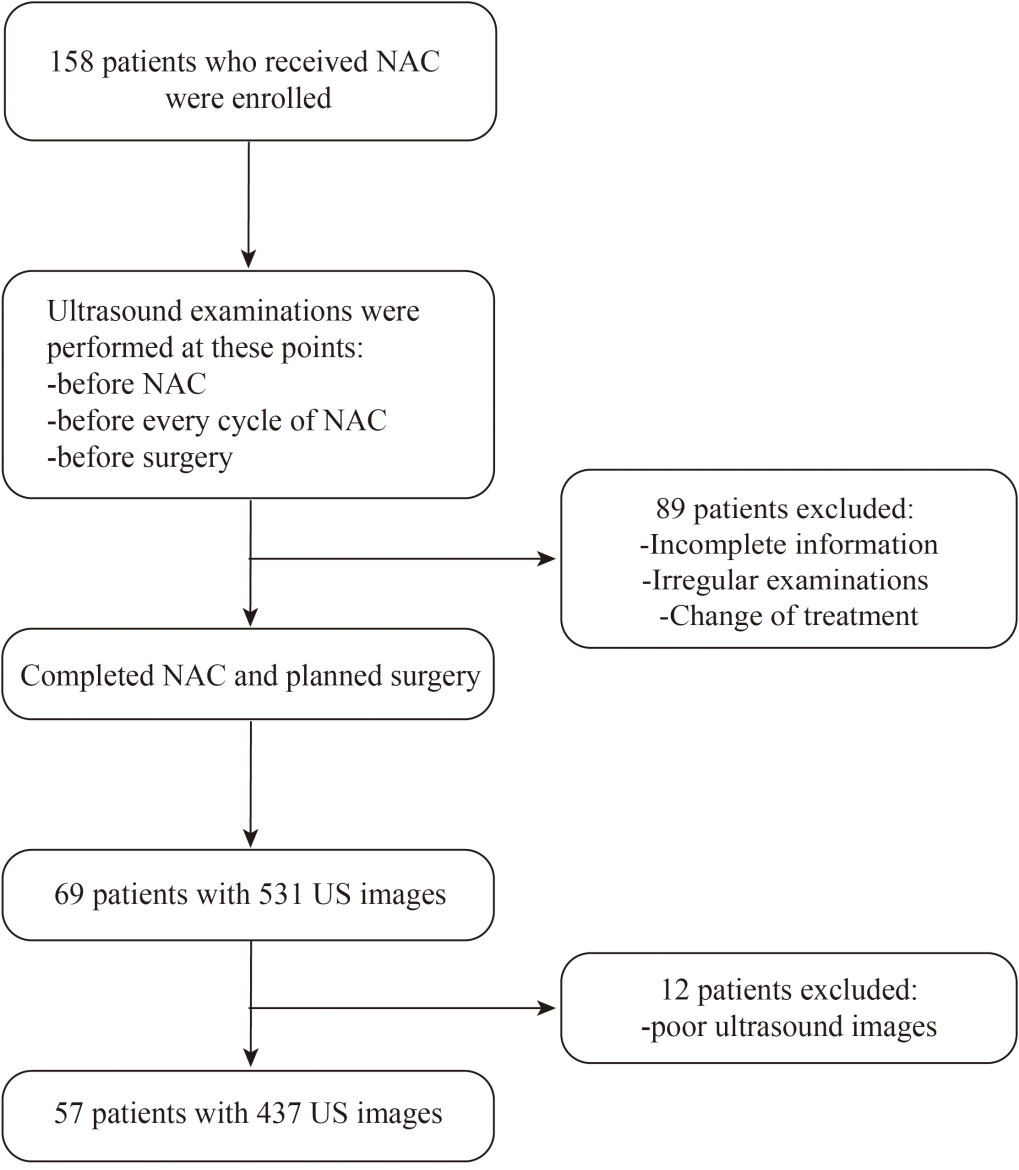
Flowchart of inclusion and exclusion for patients in the test set. US, ultrasound.

Between December 2020 and December 2022, researchers collected a total of 1393 ultrasound images from 913 patients at Renmin Hospital of Wuhan University. Among these, 956 ultrasound images from 856 patients were retrospectively collected and used as the training dataset. Additionally, 437 ultrasound images from 57 patients who underwent NAC were prospectively collected and utilized as the test dataset. This study was conducted according to the Declaration of Helsinki and relevant Chinese clinical trial research norms and regulations. Ethical approval was obtained from the Ethics Committee of Renmin Hospital of Wuhan University (approval number: WDRY2022-K217). All patients provided written informed consent for ultrasound examinations, surgical intervention, and use of data. **Figure 1** presents the patient selection flowchart.

Patient eligibility criteria included 1) a definitive diagnosis of primary invasive breast cancer by biopsy; 2) no previous treatment, and at least one of the following indications for NAC: tumor size > 5 cm; HER2 positive; estrogen receptor/progesterone receptor and human epidermal growth factor receptor (HER) 2 negative; axillary lymph node metastasis or strong breast-conserving intention; 3) underwent 6–8 cycles of complete NAC; 4) breast ultrasound examination before initiating NAC, after each NAC cycle, and after NAC completion; and 5) surgical resection after NAC completion.

Exclusion criteria were 1) patients who did not complete the NAC regimen or underwent treatment at another center; 2) breast surgery performed before NAC completion; 3) insufficient ultrasound image quality for feature extraction; and 4) lack of pathological results post-surgery.

### Neoadjuvant chemotherapy regimen

The *National Comprehensive Cancer Network guidelines for Breast Cancer (Version 3.2020)* recommend selecting the NAC regimen based on the breast cancer molecular subtype. All patients underwent 6–8 NAC cycles. The most common regimen for patients with luminal or triple-negative breast cancer includes a combination of epirubicin, cyclophosphamide, and paclitaxel, administered every 21 days. Patients with HER2-positive tumors received either the THP (paclitaxel, trastuzumab, and pertuzumab every 21 days) or TCbHP (paclitaxel, carboplatin, trastuzumab, and pertuzumab every 21 days) regimens.

### Ultrasound imaging

An ESOTE MEGAS GPX FD570A ultrasound diagnostic instrument was used for patient examination, with probe frequencies ranging 5–13 MHz. All patients were placed in the supine or side-lying position with both arms lifted and abducted to fully expose the breast. Diagnostic criteria were based on the American College of Radiology Breast Imaging Reporting and Data System. Experienced breast sonographers independently performed each ultrasonographic examination. Measurements were extracted from the ultrasound report and confirmed by another breast sonographer based on captured images. Two experienced breast sonographers manually segmented ROIs from original ultrasound images using a 3D slicer software (version 4).

### Tumor response assessment

Tumor size was assessed using ultrasound after each NAC cycle. NAC-treated tumors were also evaluated using the Response Evaluation Criteria for Solid Tumors (RECIST 1.1) (34). In this prospective study, tumor size was defined as the sum of the size of each tumor if the patient had multifocal disease. Four models were established to calculate tumor size before and after each treatment: the longest axis model (LAM), the product of two perpendicular axes model (dual-axis model [DAM]), the manual segmentation model (MSM), and the deep learning model (DLM). To compare changes in tumor size, the relative ratio was calculated after each treatment cycle using the following formula:


$$\text{Ratio N}=\frac{\text{untrasound tumor size after N cycles of NAC}}{\text{ultrsound tumor size before NAC}}$$

### Histopathological assessment

Breast cancer was diagnosed by needle biopsy of the tumor. HER2 status was assessed using immunohistochemistry and fluorescence *in situ* hybridization analysis (35). Breast tumors were classified as HER2-negative and HER2-positive subtypes. After completing NAC, surgically resected breast tumor tissue was delivered to the Department of Pathology, where a specialized breast pathologist examined the specimens to establish the pathological diagnosis. The residual cancer burden (RCB) index was used as a criterion for assessing residual tumors after NAC for breast cancer. A pCR or RCB-0 was defined as the complete absence of invasive cancer in the breast and axillary lymph nodes (8).

### Development of the deep learning-based model

Given that breast ultrasound images are characterized by low resolution and contrast and ambiguous boundaries, we utilized a custom U-Net neural network to capture tumor features by applying data augmentation (23), attention mechanism, and multi-scale method. As U-Net segments each pixel into classes, it can directly infer the ultrasound image and generate a tumor distribution map with the same dimensions as the input image. We adopted U-Net as a cancer-detection architecture and creatively added modules to construct a custom U-Net for enhancing extracted model features.

**Figure 2:**
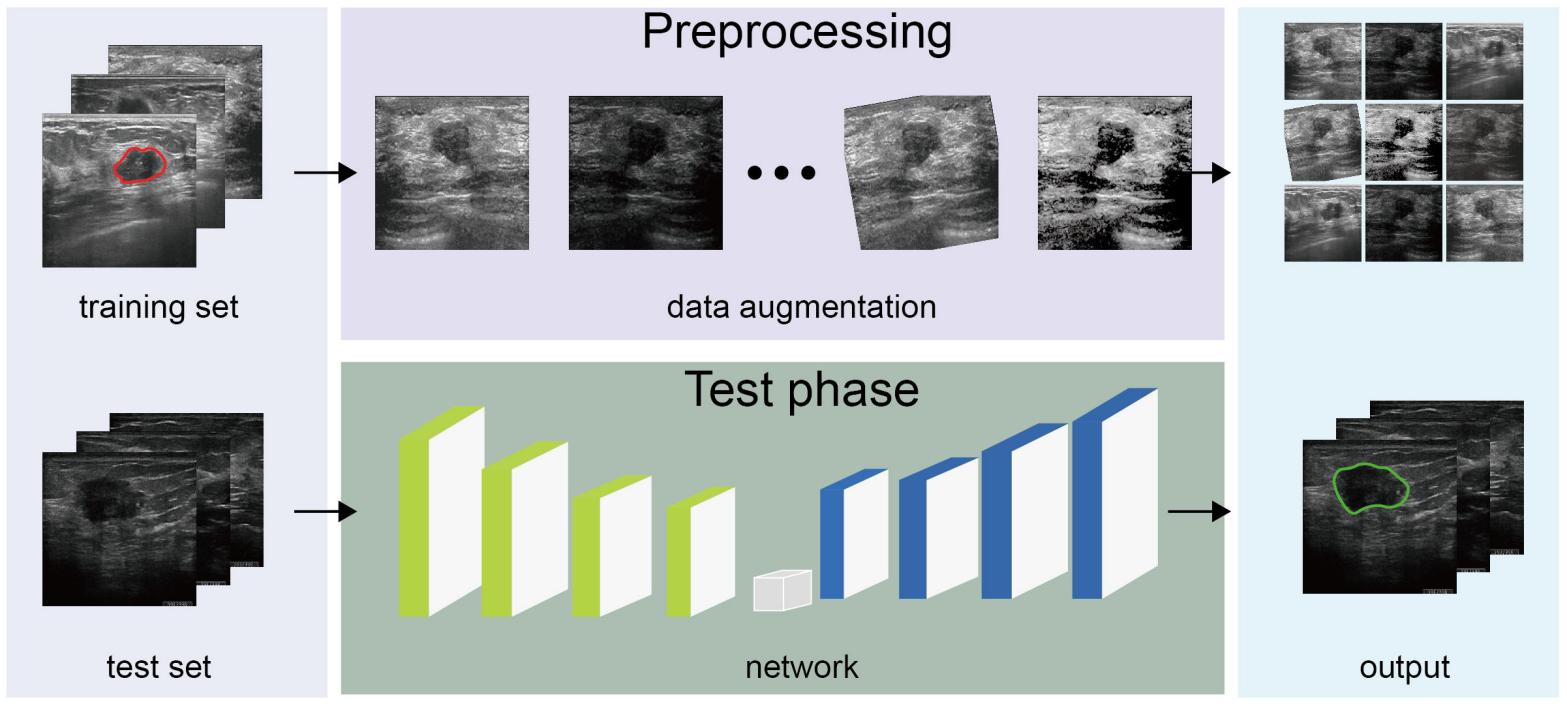
Algorithm construction Construction of the deep learning model.

**Figure 2** presents the algorithm construction process. To promote the generation and prevent overfitting, we adopted a data-augmentation technique and several network construction algorithms. During image processing, breast tumor features on the ultrasound image varied in all directions, lightness, and contrast; we utilized data-augmentation techniques such as cropping, rotation, and adjusting lighting conditions, including contrast and lightness, to extract general tumor features without intervention. We used batch normalization and dropout at each convolution layer in the neural network to avoid model oscillations and facilitate robustness. Additionally, we exploited the grayscale intensity as the input image, given that the grayscale ultrasound image contained sufficient information for the diagnosis, affording efficiency by reducing three channels into one. In the network, we introduced an attention mechanism after each convolution block, namely, the SCSE module (36), allocating more computing resources to abnormal regions and improving inference speed. Considering variations in breast tumor shape and size, a constant kernel size from the convolution layer was constrained to capture the tumor with a feasible receptive field. Therefore, we implemented multi-scale imaging to acquire different tumor sizes in ultrasound images, specifically atrous spatial pyramid pooling, with the capacity to extract multi-scale contextual information.

To ensure accurate tumor regions and contours, we proposed a hybrid loss function for model refinement. The loss function is crucial in selecting an optimizer for the model weight. We expanded the hybrid loss function for model adjustment based on the region and boundary. To obtain a more accurate intersection with the ground truth, we adopted the dice coefficient loss accompanying the binary cross-entropy loss to promote the accuracy of each pixel. Owing to the ambiguous tumor contour due to poor contrast, we adopted the active contour (37) and Hausdorff distance loss (38) to calculate the refined boundary. After applying the above techniques and changing the network, our model could precisely segment breast tumors in ultrasound images.

### Model training and model performance evaluation metrics

All datasets were collected from breast tissues examined in the ultrasound department. The dataset contains 1393 ultrasound (US) images. 956 US images of 856 patients were used as a training set to train our model, covering a variety of US images of benign breast tumors, malignant breast tumors, and post-chemotherapy breast cancer. And 437 NAC US images of 57 patients with complete NAC cycles were used as a test set to display network ability. There was no data overlap between the training set and the test set. We employed two experts to diagnose the whole dataset and generated corresponding ground truth masks. In detail, one expert annotated each image, while the other one reviewed it. When differences in diagnosis were encountered, the final annotation utilized the latter expert’s results. Meanwhile, due to variation in US image size, during network training, we rescaled the ultrasound image with a fixed resolution of 448×384 pixels. Besides, in order to quickly find the optimizer weights, we adopted the Adam algorithm (39) with the betas from 0.5 to 0.999. When conducting experiments, we set the batch size 4 learning rate of 0.002 and stopped the iteration when the model was updated for 100 epochs, saving the most accurate model parameters and avoiding overfitting. All experiments were conducted in PyTorch under an Ubuntu OS server with an Intel Xeon (R) CPU E5‐2680 v4 @2.40 GHz, 40 GB of RAM, and an NVIDIA GeForce RTX 3090 Ti with 24 GB of VRAM to boost training processing. We achieved our network on the 64-bits operation system and constructed algorithm on the Pytorch 2.0.1 framework with CuDNN 11.8. The training processing took up to 20 hours and the test phase lasted 76 seconds. Finally, the well-trained model generated a breast tumor distribution map for each ultrasound image and quantitatively analyzed performance.

To demonstrate the segmentation performance of our model, we utilized five metrics to quantitatively analyze the prediction output by comparing areas of prediction and annotation: accuracy, intersection over union (IoU), precision, recall, and the F1. All the metric formulas are show in Formula 1, 2, 3, 4, 5, and the TP, TF, FP and FN of each formula demonstrate the true positive, true negative, false positive and false negative. Among these metrics, accuracy, precision, recall, and the F1 score reflect the model’s ability to capture specific features. Specifically, accuracy showcases the correctness of pixel predictions, precision demonstrates the model’s capability to predict positive samples accurately, recall reflects the model’s ability to capture positive samples, and the F1 score provides a balanced measure considering both positive and negative samples. As for the IoU, it denotes the intersection between the predicted segmentation and the ground truth divided by the area of union, which is commonly used in segmentation tasks. Considering the definition of the IoU metric, the value belongs to 0–1, and the closer the value is to 1, the more similar the prediction to the ground truth. To automatically calculate tumor parameters, we generated geometric parameters from an ultrasound image. After processing the model, we acquired a prediction mask for the tumor region. We first annotated each image with a scale bar, which could assist in precisely calculating the geometric information. Subsequently, we calculated the tumor number, area, diameter, area ratio, and length-to-width ratio using the scale bar and prediction mask. Accordingly, the IoU metric supported the overall network performance, whereas geometric parameters indicated the breast mass condition of each breast ultrasound image.


(1) $$\text{Accuracy=}\frac{\text{TP+TN}}{\text{TP+TN+FP+FN}}$$

(2) $$\text{IOU =}\frac{\text{TP}}{\text{TP+FP+FN}}$$

(3) $$\text{Precision = }\frac{\text{TP}}{\text{TP+FP}}$$

(4) $$\text{Recall}=\frac{\text{TP}}{\text{TP}+\text{FN}}$$

(5) $$\text{F}1=\frac{2\times \text{TP}}{2\times \text{TP}+\text{FP}+\text{FN}}$$

### Statistical analysis

**Figure 3:**
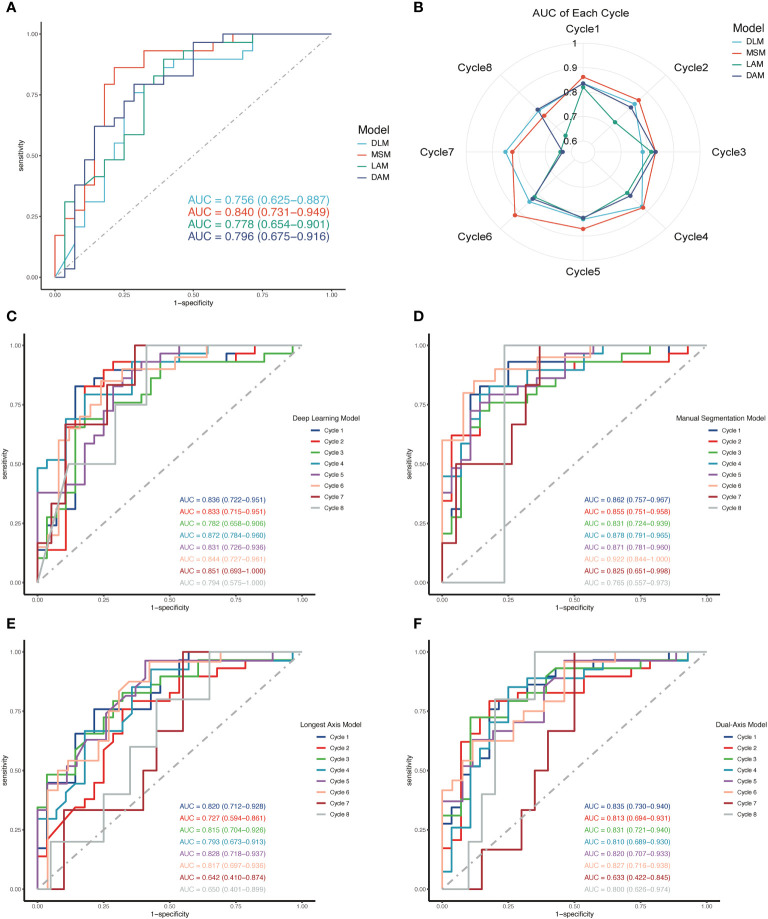
ROC curve for different models in predicting pCR. **(A)** The ROC curves were plotted to compare the ability of four different models in predicting pCR. **(B)** A radar chart shows the predictive ability of the four models at each cycle of the NAC. **(C–F)** After each cycle of NAC, tumor size was measured using four models and ROC curves were developed to predict pCR. The ROC curves for all cycles of the same model are drawn in one graph.

Statistical analyses were performed using R version 4.2.1 (R Foundation for Statistical Computing, Vienna, Austria). Normally distributed data are presented as the mean ± standard deviation. Normality was tested using the Shapiro–Wilk normality test. For comparisons between two groups, Student’s t-test was used for normally distributed data, and the Wilcoxon rank-sum test was used for non-normally distributed data. For categorical variables, the chi-square test was used to determine differences between groups. The Kruskal–Wallis method was used to compare multiple groups. By utilizing the percentage reduction of tumors relative to their initial state at a specific time point (cycle N), as the predictor variable, and considering whether pCR was achieved as the outcome indicator, we compared the predictive performance of different models by constructing receiver operating characteristic (ROC) curves (**Figure 3**). We created ROC curves for all patients after completing the entire treatment cycle using four measurement models (**Figure 3A**); after each treatment cycle using the DLM (**Figure 3C**); after each treatment cycle using the MSM (**Figure 3D**); after each treatment cycle using the LAM (**Figure 3E**); and after each treatment cycle using the DAM (**Figure 3F**). It is essential to emphasize that **Figure 3A** includes patients who completed both 6 and 8 cycles of NAC. Therefore, the ROC curve in **Figure 3A** does not overlap with any ROC curves in **Figures 3C–F**. The p values of the area under the curve (AUC) were calculated using the DeLong test. A p-value<0.05 was considered statistically significant.

## Results

### Patient information

We included 57 patients who underwent complete per-cycle ultrasound assessment for primary invasive breast cancer. **Table 1** summarizes basic patient data. The mean patient age was 49 years (range 28–70 years). Mean tumor size prior to NAC was calculated as follows: LAM 3.059 ± 1.283 cm, dual-axis model (DAM) 6.198 ± 5.734 cm^2^, manual segmentation model (MSM) 4.190 ± 3.330 cm^2^, and DLM 4.233 ± 3.638 cm^2^. Considering all patients with breast cancer, 29 (50.9%) achieved pCR after NAC and 28 (49.1%) failed to achieve pCR. The pCR and non-pCR groups differed significantly in age (p=0.020), HER2 status (p<0.001), and post-NAC tumor size (LAM, p=0.030; DAM, p=0.008; MSM, p=0.031; DLM, p=0.009).

**Table 1:** Basic patient data.

Characteristics	Patients (n=57)		p-value
	pCR (n=29)	Non-pCR (n=28)	
Age (mean ± SD) (years)	52 ± 11	47 ± 11	0.020*
Histologic type			0.862
Invasive ductal carcinoma	18	18	
Others	11	10	
Clinical N stage			0.060
cN0	9	3	
cN1-3	20	25	
HER2 status			<0.001*
HER2+	21	4	
HER2-	8	24	
Pre-NAC tumor size (mean ± SD)			
LAM (cm)	3.321 ± 1.399	2.778 ± 1.103	0.114
DAM (cm^2^)	7.133 ± 6.451	5.194 ± 4.765	0.209
MSM (cm^2^)	4.936 ± 4.261	3.479 ± 2.704	0.112
DLM (cm^2^)	4.874 ± 3.974	3.454 ± 2.316	0.135
Post-NAC tumor size (mean ± SD)			
LAM (cm)	1.148 ± 0.663	1.607 ± 0.870	0.030*
DAM (cm^2^)	0.781 ± 0.829	1.726 ± 1.647	0.008*
MSM (cm^2^)	0.549 ± 0.575	1.189 ± 1.115	0.031*
DLM (cm^2^)	0.504 ± 0.561	0.958 ± 0.936	0.009*

*p<0.05 was considered significant. NAC, neoadjuvant chemotherapy; HER2, human epidermal growth factor receptor 2; pCR, pathological complete response; LAM, the longest axis model; DAM, dual-axis model; MSM, manual segmentation model; DLM, deep learning model; SD, standard deviation.

### Performance of the DL-based model

After predicting the test-set images, we achieved a mean IoU of 0.856. Meantime, the model achieved best results on the dataset from the NAC, with an average accuracy of 0.973, average recall of 0.912 and average F1 score of 0. 918. Additionally, the segmentation capabilities of this model were quantitatively represented through ROC curve and PR curve (**Supplementary Figure S1**). The AUC for the ROC curve reached 0.99, and for the PR curve, it reached 0.92. The DLM demonstrated good discrimination ability in addressing challenges related to delineating the boundary of breast cancer ultrasound images (e.g., blurred boundary, irregular shape, uneven brightness, and interference of contrast). And the DLM could also extract breast cancer feature of ultrasound image. Simultaneously, we also tested the model’s computational complexity to display our network with more information. Our network contains 51.6 M parameters and 197.15G FLOPs and could detect a US within 0.08 seconds. In summary, the discrimination ability of the DLM was satisfactory for benign breast tumors and early-stage cancers undergoing NAC, but relatively poor for images of late-stage NAC and pCR.

### Ablation experiment

To deepen our understanding of the mechanisms of our model, we conducted an ablation experiment. Based on the functionality of the model, we divided the algorithm into three modules: U-Net, ASPP, and self-attention. We designated U-Net as the baseline, named the model with combined multi-scale ASPP as baseline-M, and the U-Net with combined self-attention module as baseline-A. All experiments were conducted using the same ultrasound images and identical data preprocessing methods to analyze the effects of each module and the overall experimental performance. Additionally, we selected four ultrasound images with different manifestations for visual analysis, providing a comprehensive examination of the algorithm’s performance from quantitative to qualitative perspectives.

In terms of cancer segmentation, the baseline model exhibited the weakest performance, with an average IoU index of only 0.742. The baseline-M, incorporating a multi-scale ASPP module, demonstrated superior performance by capturing ultrasound information at multiple scales and avoiding interference from noise or shadow areas. However, baseline-A, which integrated a self-attention mechanism into the baseline model, showed improved performance by directing more computational resources to cancer regions. Through comparative analysis, the performance gain of the baseline-A model was not as high as that of baseline-M. Nevertheless, the final model output results indicate that modules combining multi-scale and self-attention mechanisms can complement each other’s shortcomings, achieving optimal performance. Finally, the model achieved the best results on the NAC dataset, with an average accuracy of 0.973, average recall of 0.912, average IoU of 0.856, and average F1 score of 0.918 (**Table 2**).

**Table 2:** Metric results of different components on our clinical dataset from NAC.

	U-Net	ASPP	Attention	Class	Classification				Average				
					IoU	Precision	Recall	F1	Accuracy	Precision	Recall	IoU	F1
**Baseline**	**√**			**Normal**	0.952	0.958	0.993	0.975	0.955	0.926	0.78	0.742	0.835
				**Cancer**	0.531	**0.894**	0.567	0.695					
**Baseline-M**	**√**	**√**		**Normal**	0.970	0.978	0.991	0.985	0.972	**0.936**	0.886	0.842	0.909
				**Cancer**	0.714	0.893	0.781	0.833					
**Baseline-A**	**√**		**√**	**Normal**	0.962	0.977	0.984	0.981	0.965	0.903	0.875	0.812	0.888
				**Cancer**	0.661	0.829	0.766	0.796					
**Ours**	**√**	**√**	**√**	**Normal**	0.971	0.984	0.987	0.985	**0.973**	0.926	**0.912**	**0.856**	**0.918**
				**Cancer**	**0.740**	0.866	**0.836**	**0.851**					

Baseline: U-Net; Baseline-M: U-Net + Multi-scale ASPP; Baseline-A: U-Net + Self-Attention Module.

**Figure 4:**
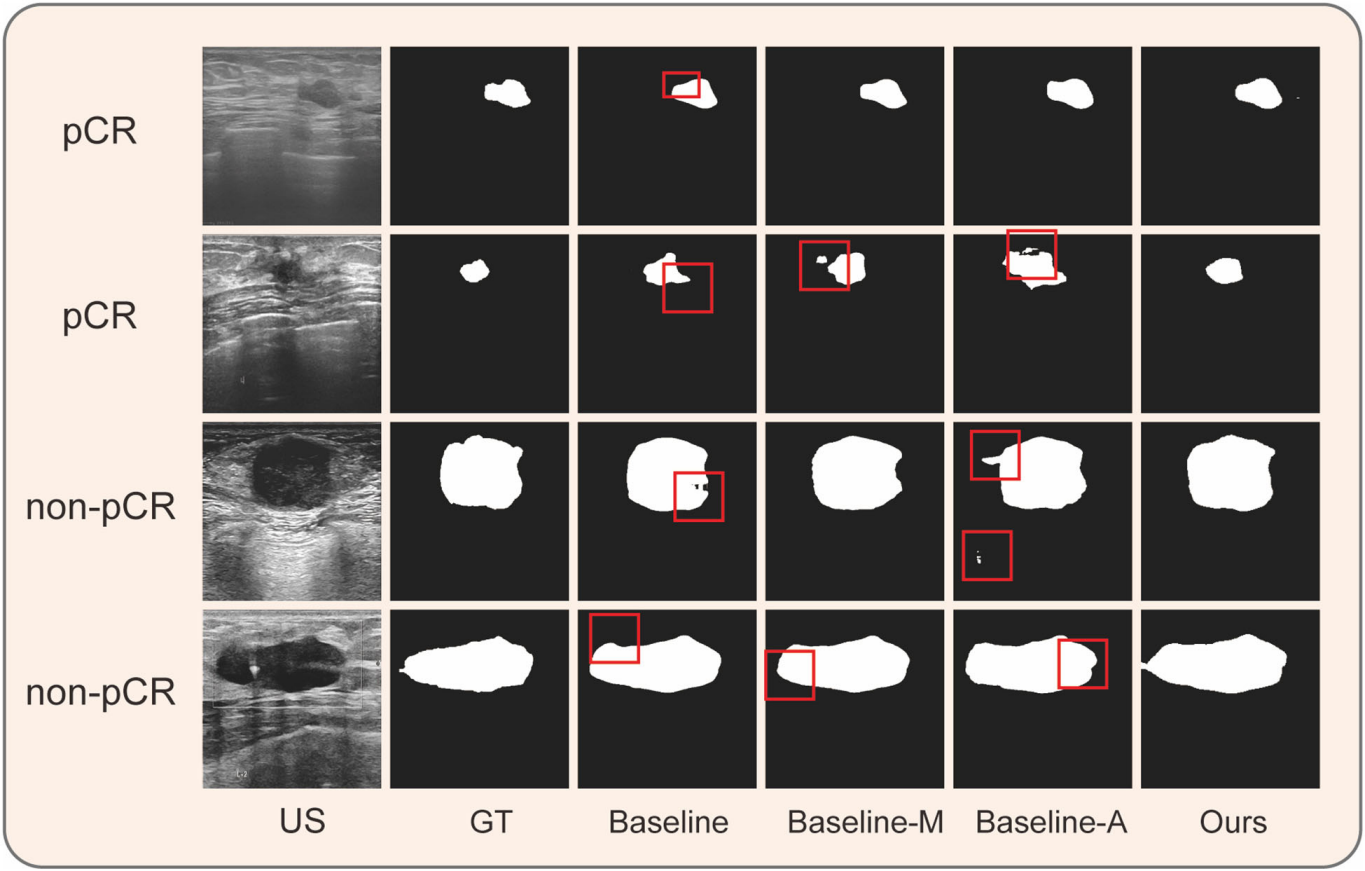
Performance of different components on four clinical ultrasound images during NAC. The results of ablation experiment. Baseline: U-Net; Baseline-M: U-Net + Multi-scale ASPP; Baseline-A: U-Net + Self-Attention Module; US, ultrasound; GT, ground truth.

Simultaneously, we visually demonstrated the performance of each model. We selected ultrasound images from four NAC treatment cycles, encompassing both pCR and non-pCR response, as well as variations in tumor boundary clarity. In **Figure 4**, we illustrated that the clearer the tumor boundary, the better the image quality, the better the model’s performance. However, in comparison to other models, our model resisted noise and shadows, accurately distinguishing the boundaries of cancer. In **Figure 4**, the baseline model exhibited the weakest resistance to interference, easily influenced by shadows and tissue around the tumor, especially in the second and third rows of the baseline images. Baseline-M, introducing a multi-scale features mechanism, significantly reduced interference from shadows and noise in ultrasound images. However, its ability to extract global information remains limited, and the accuracy of edge delineation is not precise. In the visual results of baseline-A, we observe that the attention mechanism consumes more computational resources on suspicious areas, but its detection ability is insufficient. In contrast, our proposed method, incorporating both multi-scale and self-attention mechanisms, enhances the segmentation ability to capture global information. As a result, we ultimately achieve robust cancer segmentation with improved resistance to interference.

### Measures of tumor size ratios

**Figure 5:**
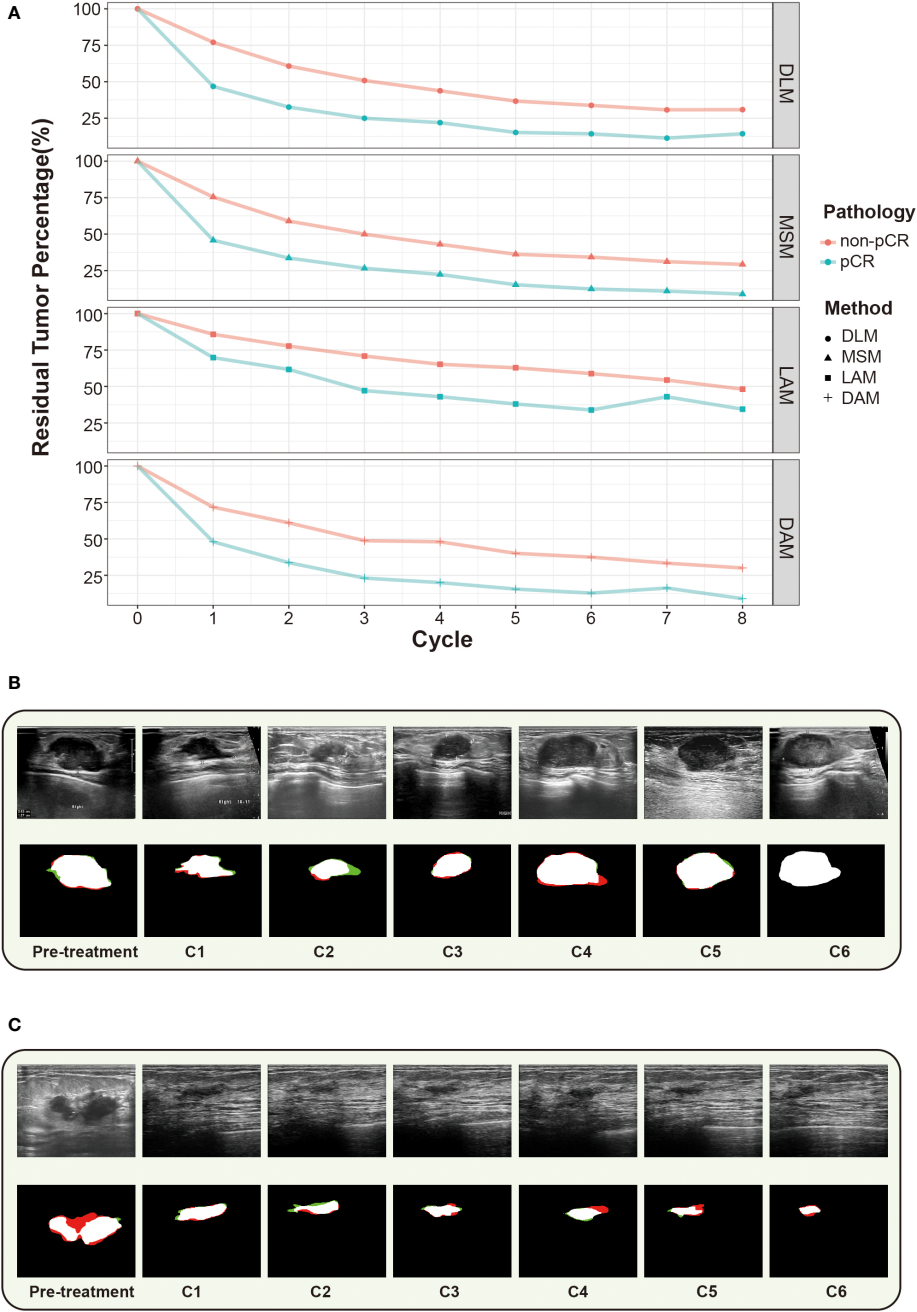
Changes in residual tumor with NAC cycles in the pCR and non-pCR groups. **(A)** The percentage of residual tumors decreased with the increase of NAC cycle in both groups, with the most evident decrease in the first two cycles. **(B)** in ultrasound images of breast cancer of a patient who didn’t receive pCR. **(C)** Changes in ultrasound images of breast cancer of a patient who received pCR. The green line denotes the prediction contour, while the red line denotes the ground truth in the first column.

Following NAC, both pCR and non-pCR groups exhibited tumor shrinkage. However, tumor regression was more significant in the pCR group than in the non-pCR group (p<0.01), particularly during the early stages of treatment (**Figures 5B, C**). As shown in **Figure 5A**, after the first two NAC cycles, the average residual tumor size measured by MSM, DLM, and DAM was <50% for patients in the pCR group, whereas the residual tumor size of patients in the non-pCR group was >50% (detailed data are displayed in the **Supplementary Files**).

### Performance of the four models for predicting pCR

ROC curves were established for the four models to predict the possibility of obtaining pCR after the NAC completion in all patients (**Figure 3A**). The AUCs for LAM, DAM, DLM, and MSM were 0.778 (95% confidence interval [CI], 0.654–0.901), 0.796 (95% CI, 0.675–0.916), 0.756 (95% CI, 0.625–0.887), and 0.840 (95% CI, 0.731–0.949), respectively. The AUCs were compared using the DeLong test (DLM vs. LAM, p=0.769; DLM vs. DAM, p=0.769; DLM vs. MSM, p=0.133; LAM vs. DAM, p=0.557; LAM vs. MSM, p=0.269; and DAM vs. MSM, p=0.358), with no significance detected (p<0.05).

Subsequently, we plotted the ROC curves for each measurement method separately across the entire course of NAC and calculated the AUC to determine which model was more powerful in predicting pCR at an early stage (**Figures 3B–F**). After the DeLong’s test, there was no significant difference between the AUCs of the different cycles (p>0.05), indicating that there was no significant difference in the predictive effect of the percentage of residual tumor size on pCR in the early (cycle 1-2), middle (cycle 3-4) and late (after 4 cycles) stages of NAC treatment. The AUC values of DLM for pCR prediction during all-phases of NAC treatment are as follows: cycle 1 (C1): 0.826; C2: 0.833; C3: 0.782; C4:0.872; C5:0.831; C6:0.844; C7: 0.851; C8:0.794 (p>0.05).

## Discussion

In this study, we collected ultrasound images of breast cancer patients underwent NAC and developed an efficient DLM for tumor detection. In comparison to various methods employed for tumor segmentation in other studies, such as He et al.’s (27) utilization of the HCT-network, Xu et al.’s (40) implementation of region attention, Lyu et al.’s (41) application of the pyramid attention, and Chen et al.’s (42) use of the cascade network, our approach stands out by efficiently extracting global and local features using the WSA module and enhancing robustness using ASSP and FAM. Our model reaches the highest IOU of 0.856 among these studies while successfully distinguishing normal tissues and mitigating noise and shadow interference. Additionally, our dataset was both extensive and diverse which collected from clinical patients with a variety of characteristics displaying the comprehensiveness of breast ultrasound images, such as age, cancer grading, and pCR status. moreover, the data size consisting of 913 patients and 1393 images, significantly exceeds that of existing public datasets, such as the 780 images in BUSI (43) and the 163 images in DatasetB (44), ensuring suitability for breast ultrasound-specific tasks. Therefore, our DLM achieving excellent segmentation performance. Herein, we employed the DLM to measure changes in tumor size during NAC and predict pathological outcomes accordingly. We aimed to assess the capability of DLM for predicting breast cancer NAC outcomes.

Firstly, we compared the sensitivity of pCR and non-pCR breast cancers to NAC by constructing tumor size change ratios and line graphs. The percentage of tumor regression in patients with and without pCR was the most notably distinguished after the first NAC cycle, and the difference in the percentage of regression between the two groups gradually decreased. Ultrasound images and DLMs have been shown to predict the treatment response in the early (cycle 1–2) or mid-treatment (cycle 4) stages of NAC (32, 33, 45). However, follow-up observations of responses to all consecutive courses of NAC are still lacking. Hence, we further explored the accuracy of predicting pCR based on the percentage of tumor regression at different treatment stages of NAC. Based on our findings, there was no significant difference in the predictive ability of ultrasound assessment for pCR when performed at any NAC cycle; therefore, ultrasound assessment can be performed at any time, regardless of the treatment course.

Furthermore, we compared the predictive abilities of the four models for pCR after NAC for breast cancer (**Figure 3**). Tumor size is known to be closely related to the therapeutic effects of NAC (11). Conventional ultrasound models such as LAM and DAM assess tumor size by measuring dimensions. MSM and DLM assess tumor size by measuring area. The constructed DLM possesses the same level of capability as experienced sonographers in accurately identifying tumor boundaries. There was no significant difference in the predictive efficacy of the four models for pCR. Therefore, the DLM can serve as a valuable tool for assisting sonographers in manually measuring breast cancer, enabling more precise calculations of tumor size. Notably, our DLM is still helpful in clinical practice. First, it helps improve the workflow. The algorithms rapidly and automatically identify tumor areas within ultrasound images, allowing for precise segmentation and accurate tumor size measurements. The DLM developed in this study offers a time-efficient model to reduce the burden on the sonographers and eliminate bias due to differences in experience. The predictive effect of the DLM is also relatively stable throughout the treatment process compared to conventional measurement models (**Figure 3B**). Second, our DLM was learned from the database of a large general hospital and reviewed by senior sonographers, and the DLM’s ability to recognize ultrasound images is equivalent to that of an experienced sonographer. Therefore, the DLM is useful for assisting junior sonographers or primary hospitals in breast ultrasound examinations. Third, our research helps doctors and patients by facilitating the tracking of tumor growth and treatment responses. It enables the recording of tumor size and shape at different time points, allowing for image comparisons. This automatic cancer detection capability enables clinicians to evaluate treatment effectiveness and adjust treatment plans, as necessary. Most importantly, predicting tumor responses and tailor personalized treatment plans to ensure the best treatment outcomes for patients.

The direct comparison of our results with those of other studies can be challenging, given the differences in data collection and analysis methods. To the best of our knowledge, this is the first study to use DL to continuously monitor changes in tumor size during NAC. The previous studies similar to this research and their AUC values for predicting pCR have been listed in **Table 3**. Candelaria et al. (45) and Gounaris et al. (46) predicted pCR by measuring the largest change in tumor diameter at mid-treatment. Sannachi et al. (47) employed quantitative ultrasonography to collect data at the 1^st^, 4^th^, and 8^th^ NAC cycles. Byra et al. (32) used a DL approach based on early treatment ultrasound images to assess the treatment response. The authors also performed consecutive predictions during the first four cycles, concluding the absence of any significant difference in the prediction of chemotherapy outcomes during the first four cycles (48), consistent with our findings.

**Table 3:** Similar studies.

Studies	AUC
Candelaria et al.	0.760 for patients with TNBC 0.822 for HR+/HER2- patients 0.522 for HR-/HER2+ patients
Gounaris et al.	0.689
Byra et al.	0.797
Sannachi et al.	0.780-0.860

AUC, area under the curve; TNBC, triple-negative breast cancer; HER2, human epidermal growth factor receptor 2.

In the present study, the following innovations are pivotal. First, the DL method for processing ultrasound images has several advantages. 1) Our DL method based on U-Net is an automatic end-to-end neural network that does not manually screen handcrafted features. The model can infer the entire ultrasound image and provide prediction results directly. Moreover, the model can distinguish the tumor boundary from breast tissue by intelligently extracting morphological features from cancer and normal tissues. Meanwhile, the prediction image had the same dimensions as the input image, displaying breast cancer distribution. 2) Our model adopted a multi-scale method and attention mechanism to improve the ability to extract and focus on features for accurate and rapid tumor tissue identification. Moreover, the multi-scale method considers context information to improve boundary performance, particularly the relationship between the tumor and normal tissue region. 3) To enhance the detection capacity, we adopted a hybrid loss function to search for optimal model parameters. By improving the accuracy of the tumor contour and pixel segmentation, our hybrid loss function consists of binary cross-entropy loss, dice loss, and active contour models. Second, to the best of our knowledge, this is the first study to evaluate ultrasound findings during NAC. Herein, the ultrasound assessment values were similar for each cycle. Third, we compared the effectiveness of conventional ultrasound tumor measurements and DLM in predicting pathologic response, with the DLM as an alternative to manual measurements.

Nevertheless, the limitations of the present study need to be addressed. First, this was a single-center study. Although this study was conducted over 2 years, recruiting an adequate number of representative patients was challenging. Further external validation should be performed by recruiting more patients in a multicenter prospective study to demonstrate the accuracy of the DLM. Second, the DLM predictions were solely based on changes in tumor size. Although size change is the most important and intuitive indicator of tumors’ response to chemotherapy, this approach overlooks other ultrasound image features related to the response to NAC, such as texture changes, tumor shrinkage patterns, and lymph node status. Including these additional features may further enhance the predictive performance of the model. Third, although ultrasound is one of the most important imaging modalities for breast cancer NAC, predictions based on ultrasound images alone are insufficient to determine whether the NAC treatment regimen should be stopped or changed. Many guidelines suggest employing dynamic contrast enhanced (DCE) MRI for assessing the efficacy of NAC. However, this study lacks a comparative analysis between ultrasound and MRI. Therefore, the clinical value of the DLM based on breast ultrasound for pCR outcome prediction remains to be validated.

Future research will be primarily focused on several key areas. First, there should be a focus on the multimodal fusion of medical imaging data to provide a more comprehensive medical insight for predicting the tumor’s response. Second, there is a need for development of automated annotation tools to alleviate the burden of data labeling for medical professionals and researchers. Third, there should be a quantification of uncertainty and research into interpretability, particularly in medical ultrasound imaging. Fourth, there should be a focus on incremental learning to enable continuous adaptation to new data, with a special emphasis on monitoring long-term tumor changes and addressing new tumor types. These research directions will help enhance the efficiency and accuracy of medical image analysis, fostering ongoing improvements in tumor diagnosis and treatment.

## Conclusions

In conclusion, we constructed a U-Net-based end-to-end DLM for processing ultrasound images during NAC treatment for breast cancer, which offers advantages of accuracy, efficiency, and automation to assist manual measurement. This study provides a non-invasive method for predicting individualized responses in breast cancer patients undergoing NAC at all stages of treatment.

## Data availability statement

The original contributions presented in the study are included in the article/**Supplementary Material**. Further inquiries can be directed to the corresponding authors.

## Ethics statement

The studies involving humans were approved by the Ethics Committee of Renmin Hospital of Wuhan University (approval number: WDRY2022-K217). The studies were conducted in accordance with the local legislation and institutional requirements. The participants provided their written informed consent to participate in this study.

## Author contributions

JZ: Resources, Writing – original draft. JD: Formal Analysis, Writing – original draft. JH: Formal Analysis, Writing – original draft. LM: Methodology, Writing – review & editing. NL: Resources, Writing – review & editing. FY: Supervision, Writing – review & editing. CL: Methodology, Project administration, Writing – review & editing. SS: Supervision, Writing – review & editing. YZ: Funding acquisition, Project administration, Supervision, Writing – review & editing.
