# Supplementary material for: Monitoring response to neoadjuvant therapy for breast cancer in all treatment phases using an ultrasound deep learning model
Source: Front Oncol. 2024 Jan 24;14:1255618. doi: 10.3389/fonc.2024.1255618 (PMC10847543; doi:10.3389/fonc.2024.1255618)
Supplement: Supplementary file 1 [file DataSheet_1.docx]

**Supplementary files**

Table S1. Comparison of tumor size ratios in the pCR and non-pCR groups for each cycle of NAC treatment measured by four models

| Tumor size ratios  (Mean ± SD) | pCR (n=29) | Non-pCR (n=28) | P values |
| --- | --- | --- | --- |
| The longest axis model |  |  |  |
| Ratio 1 | 0.693±0.155 | 0.902±0.181 | <0.001* |
| Ratio 2 | 0.639±0.255 | 0.826±0.342 | 0.003* |
| Ratio 3 | 0.488±0.187 | 0.743±0.286 | <0.001* |
| Ratio 4 | 0.468±0.201 | 0.726±0.319 | <0.001* |
| Ratio 5 | 0.401±0.189 | 0.697±0.308 | <0.001* |
| Ratio 6 | 0.366±0.147 | 0.635±0.251 | <0.001* |
| Ratio 7 | 0.429±0.126 | 0.574±0.273 | 0.315 |
| Ratio 8 | 0.344±0.157 | 0.504±0.247 | 0.324 |
| Dual-axis model |  |  |  |
| Ratio 1 | 0.450±0.194 | 0.802±0.321 | <0.001* |
| Ratio 2 | 0.350±0.275 | 0.703±0.601 | <0.001* |
| Ratio 3 | 0.238±0.185 | 0.537±0.417 | <0.001* |
| Ratio 4 | 0.218±0.189 | 0.567±0.720 | <0.001* |
| Ratio 5 | 0.171±0.157 | 0.468±0.343 | <0.001* |
| Ratio 6 | 0.145±0.110 | 0.418±0.329 | <0.001* |
| Ratio 7 | 0.163±0.070 | 0.354±0.339 | 0.355 |
| Ratio 8 | 0.090±0.043 | 0.327±0.283 | 0.042* |
| Manually segmentation model |  |  |  |
| Ratio 1 | 0.437±0.192 | 0.778±0.353 | <0.001* |
| Ratio 2 | 0.315±0.218 | 0.649±0.324 | <0.001* |
| Ratio 3 | 0.248±0.166 | 0.566±0.434 | <0.001* |
| Ratio 4 | 0.194±0.128 | 0.516±0.320 | <0.001* |
| Ratio 5 | 0.153±0.105 | 0.429±0.253 | <0.001* |
| Ratio 6 | 0.110±0.089 | 0.402±0.256 | <0.001* |
| Ratio 7 | 0.139±0.076 | 0.358±0.276 | 0.017* |
| Ratio 8 | 0.132±0.048 | 0.329±0.209 | 0.120 |
| Deep learning model |  |  |  |
| Ratio 1 | 0.406±0.178 | 0.738±0.331 | <0.001* |
| Ratio 2 | 0.279±0.179 | 0.574±0.286 | <0.001* |
| Ratio 3 | 0.266±0.250 | 0.523±0.380 | <0.001* |
| Ratio 4 | 0.178±0.145 | 0.488±0.253 | <0.001* |
| Ratio 5 | 0.133±0.099 | 0.403±0.265 | <0.001* |
| Ratio 6 | 0.108±0.117 | 0.385±0.268 | <0.001* |
| Ratio 7 | 0.094±0.078 | 0.325±0.274 | 0.009* |
| Ratio 8 | 0.093±0.113 | 0.267±0.185 | 0.080 |

*p<0.05 was considered statistically significant.


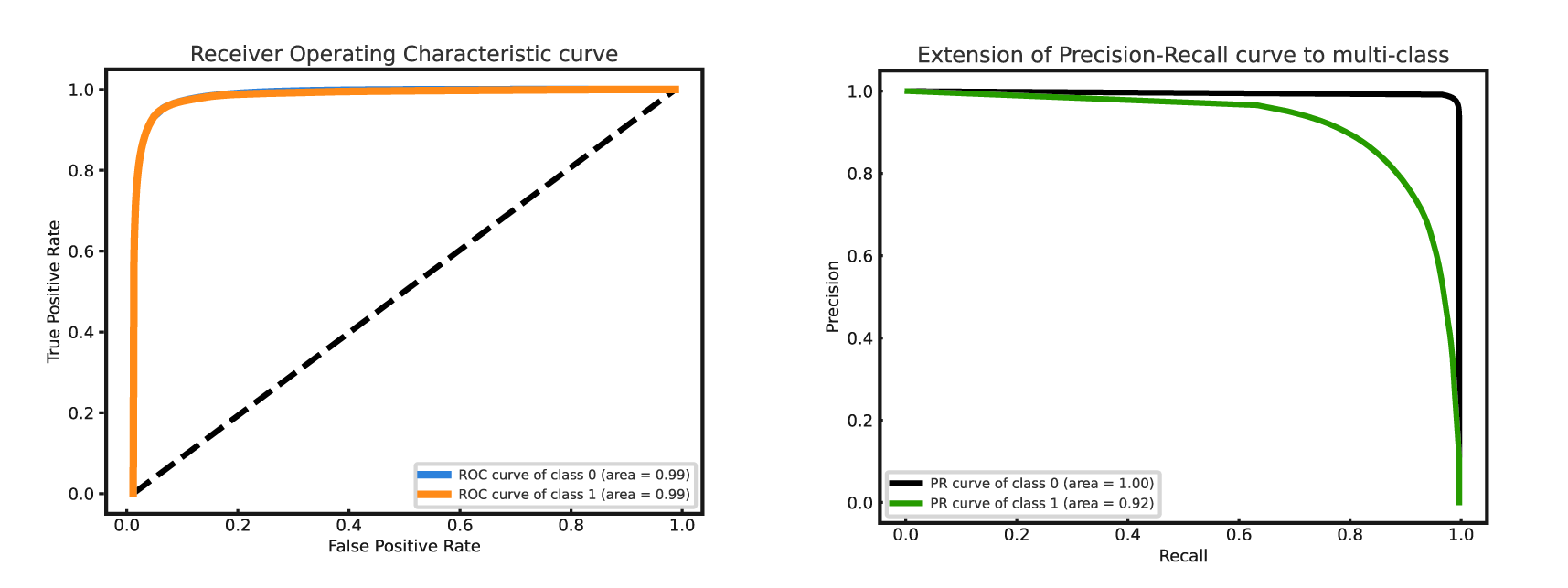


Figure S1. The ROC curve and PR curve of the segmentation capabilities of DLM
